# Effects of Variable Resistance Training Within Complex Training on Neuromuscular Adaptations in Collegiate Basketball Players

**DOI:** 10.2478/hukin-2022-0094

**Published:** 2022-11-08

**Authors:** Lin Shi, Mark Lyons, Michael Duncan, Sitong Chen, Zhenxiang Chen, Wei Guo, Dong Han

**Affiliations:** 1 School of Physical Education and Sport Training, Shanghai University of Sport, Shanghai, China; 2 Sport and Human Performance Research Centre, University of Limerick, Limerick, Ireland; 3 Centre for Sport, Exercise and Life Sciences, Coventry University, Coventry, UK; 4 Institute for Health and Sport, Victoria University, Melbourne, Australia

**Keywords:** elastic band, strength, jump, sprint, postactivation performance enhancement

## Abstract

The aim of this study was to investigate the differences in neuromuscular performance between variable resistance training and constant resistance training within complex training. Twenty-one well-trained collegiate basketball players were randomly assigned to either an experimental group (variable resistance training) or a control group (constant resistance training) and completed a twice weekly training program over an 8-week period. Training programs were the same except that the experimental group included variable resistance via elastic bands (40% of the total load). Maximum strength, vertical jump, horizontal jump, and sprint performance were assessed pre- and post-intervention. Both groups demonstrated significant increases in the back squat 1RM (experimental group +36.5% and control group +32.3%, both p < 0.001), countermovement jump (experimental group +12.9%, p = 0.002 and control group +5.6%, p = 0.02), and squat jump performance (experimental group +21.4% and control group +12.9%, both p < 0.001), whereas standing broad jump performance improved only in the experimental group (+2.9%, p = 0.029). Additionally, the experimental group showed significant improvement in the squat jump (p = 0.014) compared with the control group. However, no statistically significant differences were found between groups for countermovement jump (p = 0.06) and sprint performance at 10 m (p = 0.153) and 20 m (p = 0.076). We may conclude that both training modalities showed similar improvements in maximum strength. Performing variable resistance training within a complex training program is more efficient to enhance selective power performance in well-trained collegiate basketball players.

## Introduction

Basketball is a team sport that comprises high levels of powerful physical attributes such as jumping and sprinting (Schelling and Torres-Ronda, 2016). The ability to perform such actions requires optimal combination of force and velocity, and therefore producing maximal power output which is a crucial determinant in basketball. Resistance training is broadly used to develop muscular strength and power (Suchomel et al., 2018). Typically, the external load is constant throughout the range of motion of these exercises. However, constant resistance training (CRT) may not be the most conducive means to elicit maximal musculature activation as a result of mechanical disadvantages at specific joint angles (Kompf and Arandjelović, 2016). Alternatively, variable resistance training (VRT) where elastic bands are combined with free weights can vary the external load across the entire range of motion (Frost et al., 2010). Such a loading pattern is considered to greatly accommodate the exercises with an ascending strength curve, such as the back squat (Wallace et al., 2018). Some studies examining acute neuromuscular responses have demonstrated that VRT significantly increases muscle activation, peak force, and peak power during back squats compared with CRT (Andersen et al., 2016; Wallace et al., 2006). Consequently, VRT has been proposed as an efficacious loading modality to optimize neuromuscular adaptations (i.e., force and velocity).

A recent meta-analysis revealed that VRT and CRT lead to similar gains in maximum strength (*p* = 0.88) (Nilo dos Santos et al., 2018). The different VRT methodologies may be an important consideration when interpreting studies on this topic, such as the contribution of variable resistance and loading schemes (Wallace et al., 2018). Furthermore, the findings with respect to power performance are also equivocal. Despite some studies supposing the superiority of VRT in terms of velocity-specific adaptation (Frost et al., 2010; Wallace et al., 2018), several studies have shown no significant differences between VRT and CRT (Andersen et al., 2015; Ataee et al., 2014; Katushabe and Kramer, 2020). The lack of powerful tasks within the intervention may result in the unchanged power outcomes. Recently, some studies demonstrated the benefit of acute power increases following VRT by inducing postactivation performance enhancement (PAPE) (Krčmár et al., 2021; Mina et al., 2019). Typically, PAPE proposes that a high-intensity conditioning activity induces a performance-enhancing physiological mechanism previously called postactivation potentiation (PAP) (Blazevich amd Babault, 2019). A number of potential mechanisms included, but is not limited to, increased phosphorylation of the myosin light chain, increases in muscle temperature, increases in muscle blood flow/water content and increased muscle activation (Blazevich and Babault, 2019). In practical settings, strength and conditioning practitioners commonly prescribe complex training by manipulating PAPE mechanisms to further improve power performance (e.g., a set of back squats followed by a set of plyometrics) (Cormier et al., 2020; Martínez-García et al., 2021; Pagaduan and Pojskic, 2020). Therefore, it is conceivable that utilizing VRT within a complex training program might be more effective in enhancing power adaptation. However, to date, research examining the long-term effect of complex training using VRT is scant and warrants further investigation.

Previous research has utilized different VRT methodologies, and understanding acute neuromuscular responses may provide insight into how to most effectively manipulate VRT strategies (Frost et al., 2010). Andersen et al. (2016) found that a high contribution of variable resistance (81% of the total load) produced higher muscle activity compared to a medium contribution of variable resistance (49% of the total load). Given that muscle activation partly underpin PAPE (Tillin and Bishop, 2009), it might be hypothesized that a higher contribution of variable resistance in VRT may better potentiate subsequent power performance. This notion is somewhat supported in the research whereby a 30% VRT condition (30% of the total load (85% 1-repetition maximum (RM)) induced a greater PAPE in selective power outcomes than 20% VRT conditions (Krčmár et al., 2021). However, another study using similar loading reported no significant between-group differences in all posttests (Wyland et al., 2015). The disparity here could be partly attributed to the loading scheme used in VRT where the loading at the top was equal (85% 1RM) between VRT and CRT (Wyland et al., 2015), therefore a relatively lower intensity in VRT may be insufficient to elicit greater PAPE. In light of the above consideration, the current study utilized a high contribution of variable resistance and a relatively equivalent loading scheme to investigate the longitudinal effects of VRT and CRT within a complex training intervention on neuromuscular adaptations. We hypothesized that VRT would elicit greater adaptations in maximum strength and power-related performance compared with CRT.

## Methods

### Participants

Twenty-one well-trained collegiate male basketball players (10 guards, 9 forwards and 2 centres) volunteered to participate in this study (mean ± SD; age 20.8 ± 1.4 years; stature 186.3 ± 7 cm; body mass 82.8 ± 12.8 kg). A total sample size of at least 16 participants was determined following a power calculation for 85% statistical power, an alpha error of 0.05 and an effect size (ES) of 0.75 (Arazi et al., 2020). Inclusion criteria for participation were actively engaged in basketball training and competition, and no physical limitation and health issues that could affect testing and training. All participants were at least certified national II level of performance in basketball, with an average of 6 years of prior basketball training experience. In the 6 months prior to the intervention, players routinely performed a minimum of 2 × 120 min basketball training sessions (including technical, tactical and conditioning elements) per week.

All participants were informed of the intervention procedures, the potential benefits and risks, and all signed written informed consent in advance of the study. The study was approved by the Shanghai University of Sport Science Research Ethics Committee (ID number:102772021RT086). The study conformed with the ethical standards of the Helsinki Declaration.

### Measures

#### Elastic bands tension measurement

Elastic bands (Rising, Nantong, China) were anchored to the dumbbells and looped over the unloaded barbell. The participant stood on a force plate (Kistler, model 9290AA, Winterthur, Switzerland), the mass of the player and the barbell were accounted for. Resistance originated from elastic bands was adjusted to 40% of the total load at 85% 1RM when the participant was standing, while bands were slack at the full squatting position, thereby no loading provided. Based on participant’s 1RM data, appropriate elastic bands (blue, yellow, pink) were then selected to achieve the intended loading. The actual measured elastic bands tension was 2.15 ± 2.11% and 33.46 ± 2.59% of the 1RM at the bottom and the top.

#### Maximum strength test

The test was performed in a power rack (Hammer, Rosemont, USA). After a general warm-up, participants performed three sets of 50% (7−10 repetitions), 70% (5−7 repetitions), and 80% (3−5 repetitions) estimated 1RM with 2 min rest intervals between sets. The warm-up load was quantified using the estimated 1RM load based on their body mass and training experience. After the final warm-up set, players performed 3 to 4 trials at their estimated 3RM, separated by 4 min recovery. Based on the formula by NSCA guidelines (Haff and Triplett, 2016), 1RM was calculated.

#### 20m sprint (with 10 m split time) test

Three pairs of timing gates (Smart Speed; Fusion Sport, Brisbane, Australia) were placed at 0 m, 10 m and 20 m. Participants stood 0.2 m behind the photocell beam, with a staggered position. Two trials separated by 2 min recovery were conducted on an indoor running track. The fastest time was recorded. The intraclass correlation coefficient (ICC) was 0.87 (95% CI: 0.70−0.95) for 20 m.

#### Vertical jump test

The vertical jump tests consisted of the countermovement jump (CMJ) and squat jump (SJ) following the previous protocols (Markovic et al., 2004). Three trials with 2 min recovery were completed for each jump and the greatest performance was used in further analysis. Two submaximal practice trials with 1 min of recovery were performed before each jump test. To perform the CMJ, participants began from an upright position with hands on their hips, rapidly executing a downward movement of self-determined depth followed by a vertical maximum height jump, keeping legs straight throughout. For the SJ, players were instructed to hold a static squat position with 90° knee flexion for 3 s before jumping. The jump height was calculated from the flight time data derived from a jump mat (Smart Jump; Fusion Sport, Brisbane, Australia). ICCs for the CMJ and the SJ were 0.94 (95% CI: 0.87−0.98) and 0.96 (95% CI: 0.90−0.98), respectively.

#### Standing broad jump test

The standing broad jump (SBJ) was performed on the indoor running track along the side of a measuring tape. Based on a previous testing protocol (Markovic et al., 2004), participants started with their toes on a 0 cm marked line, and using the arm swing jumped as far as possible. A practice trial at sub-maximal effort was performed before testing. Then, three maximal SBJ trials were conducted, with 2 min rest intervals between trials. The ICC was 0.92 (95% CI: 0.82−0.96).

### Design and procedures

A mixed design exploring both within- and between-groups differences was used to compare the effects of VRT and CRT within a complex training program on maximum strength, CMJ, SJ, SBJ and 20 m sprint performance. Participants were matched in terms of pre-intervention strength data and then were randomly separated into either the VRT group (n = 11) or the CRT group (n = 10).

**Figure 1:**
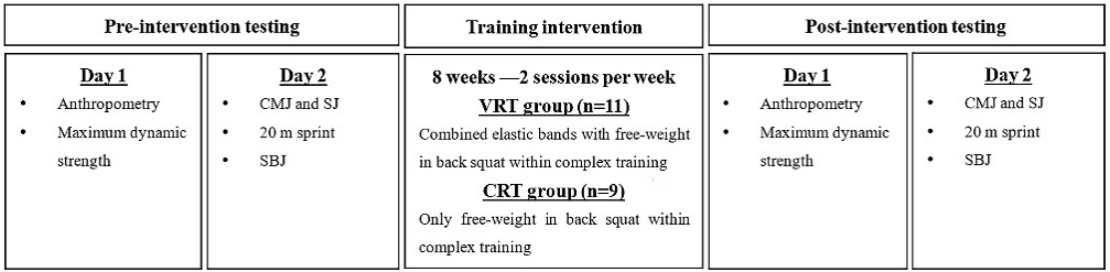
Timeline of testing and the intervention. VRT: variable resistance training; CRT: constant resistance training; CMJ: countermovement jump; SJ: squat jump; SBJ: standing broad jump

Prior to the commencement of the pre-test, all participants completed 3 sessions to familiarize themselves with the back squat, plyometric tasks, and the testing procedures. After the familiarization sessions, participants conducted a series of physical performance evaluations on two testing occasions. On Monday, anthropometry was evaluated prior to the maximum strength test. On Thursday, the tests were performed in the following order: (1) CMJ and SJ; (2) 20 m sprint; (3) SBJ. A 10-15 min rest interval was provided between each test. The day after the maximum strength test was performed, in the VRT group the specific contribution of variable resistance was measured via a force plate, and then players were familiarized with VRT. Following the 8-week intervention, all participants completed post-test in the same order as in the pre-test (Figure 1). Prior to each session, all participants performed a general warm-up comprising 5 min of running at low intensity, followed by 5–10 min of lower limb dynamic stretching and activation drills.

### Training program

Participants trained two days per week for a period of 8 weeks, each session lasted 60 min. Complex training consisted of a set of back squats followed by a set of plyometrics (Table 1). Within each complex pair, a 3 min rest interval was allowed because a similar interval had been demonstrated to elicit an optimal PAPE effect (Mina et al., 2019). In addition, a 4 min rest interval was included between complex pairs since 2 to 5 min interset recovery is suggested to produce the greatest strength benefits (Suchomel et al., 2018). The back squat 1RM value was reassessed in the mid-intervention and the load was updated accordingly.

**Table 1:** Details of the 8-week complex training program.

Week 1–2 (set × repetitions/%1RM)	Week 3–4 (set × repetitions/%1RM)	Week 5–6 (set × repetitions/%1RM)	Week 7–8 (set × repetitions/%1RM)
	Back squat (1 × 4/85)	Back squat (1 × 4/85)	
Back squat (1 × 6/80)	45 cm DJ (1 × 4)	60 cm DJ (1 × 3)	Back squat (1 × 3/90)
CMJ (1 × 5)	Back squat (1 × 4/85)	Back squat (1 × 4/85)	CMJ (1 × 5)
Back squat (1 × 6/80)	CMJ (1 × 5)	CMJ (1 × 5)	Back squat (1 × 3/90)
30 cm DJ (1 × 5)	Back squat (1 × 4/85)	Back squat (1 × 4/85)	45 cm DJ (1 × 4)
Back squat (1 × 6/80)	45 cm DJ (1 × 4)	60 cm DJ (1 × 3)	Back squat (1 × 3/90)
SBJ (1 × 5)	Back squat (1 × 4/85)	Back squat (1 × 4/85)	SBJ (1 × 5)
Back squat (1 × 6/80)	SBJ (1 × 5)	SBJ (1 × 5)	Back squat (1 × 3/90)
20 m sprint (1 × 2)	Back squat (1 × 4/85)	Back squat (1 × 4/85)	20 m sprint (1 × 2)
	20 m sprint (1 × 2)	20 m sprint (1 × 2)	

*RM: repetition maximum; CMJ: countermovement jump; SBJ: standing broad jump; DJ: drop jump.*

To ensure similar loading across both groups, half of the variable resistance (17% 1RM) was removed from the free-weight in the VRT group, as previously reported (Krčmár et al., 2021; Mina et al., 2019). In addition, when adjusting the intensity of the back squat throughout the intervention, the contribution of variable resistance remained constant and only the free-weight varied in the VRT group (Andersen et al., 2015). All training sessions were fully supervised by two researchers. Participants were required to refrain from any other lower body strength training during the intervention.

### Statistical analysis

Descriptive statistics (mean ± SD) were carried out using SPSS 25.0 (IBM SPSS Inc., Chicago, IL, USA). All data were examined for normality and homogeneity via Shapiro-Wilk and Levene’s tests, respectively. To examine group × time interactions and within- and between-group differences, a 2 (groups: VRT vs. CRT) × 2 (time: pre-intervention vs. post-intervention) repeated-measures ANOVA with Bonferroni adjustment was used to analyze every outcome variable using the mean difference with a corresponding confidence interval (95% CI). Anthropometric characteristics and pre-intervention values were evaluated using one-way ANOVA. Percentage changes were calculated as ([post-pre]/pre × 100). ES was calculated using Hedge’s *g* (Borenstein et al., 2011), with the interpretation of ES as follows: < 0.35, 0.35 to 0.8, 0.8–1.5, and > 1.5 for trivial, small, moderate, and large ES, respectively (Rhea, 2004). Statistical significance was set at *p* < 0.05.

## Results

One participant withdrew from the CRT group due to injury not related to the intervention. There were no significant differences in any performance variable at the pre-intervention (*p* > 0.05). The mean values and changes in the performance assessment are displayed in Table 2.

**Table 2:** Comparison of strength and power performance assessment for within- and between-group (mean ± SD)

Assessment	Intervention	Pre	Post	Δ(%)	*p* value	ES
1RM (kg)	VRT	123.18 (27.67)	168.09 (27.12)	36.5%	< 0.001	1.58 (Large)
	CRT	130.67 (14.84)	172.89 (16.08)	32.3%	< 0.001	2.60 (Large)
	Δ(%)	-6.1%	-2.9%			
	*p* value	0.476	0.646			
	ES	0.31	0.2			
CMJ (cm)	VRT	48.96 (6.33)	55.29 (8.34)	12.9%	0.002	0.82 (moderate)
	CRT	46.03 (5.29)	48.60 (6.10)	5.6%	0.02	0.43 (small)
	Δ (%)	6%	12.1%			
	*p* value	0.283	0.06			
	ES	0.48	0.86			
SJ (cm)	VRT	41.81 (4.13)	50.76 (6.06)	21.4%	< 0.001	1.66 (Large)
	CRT	39.40 (3.96)	44.47 (3.68)	12.9%	< 0.001	1.26 (moderate)
	Δ (%)	6.1%	14.1%			
	*p* value	0.202	0.014			
	ES	0.57	1.17			
SBJ (cm)	VRT	268.36 (16.42)	276.27 (20.91)	2.9%	0.029	0.40 (small)
	CRT	256.22 (19.01)	261.44 (16.99)	2%	0.094	0.28 (trivial)
	Δ (%)	4.7%	5.7%			
	*p* value	0.143	0.104			
	ES	0.66	0.74			
10 m sprint (s)	VRT	1.73 (0.08)	1.72 (0.07)	-0.6%	0.466	-0.13 (trivial)
	CRT	1.78 (0.09)	1.77 (0.07)	-0.6%	0.689	-0.12 (trivial)
	Δ (%)	-2.8%	-2.8%			
	*p* value	0.213	0.153			
	ES	-0.57	-0.68			
20 m sprint (s)	VRT	3.01 (0.13)	3.00 (0.12)	-0.3%	0.709	-0.08 (trivial)
	CRT	3.10 (0.13)	3.11 (0.14)	0.3%	0.729	0.07 (trivial)
	Δ (%)	-2.9%	-3.5%			
	*p* value	0.161	0.076			
	ES	-0.66	-0.81			

*RM: repetition maximum; CMJ: countermovement jump; SJ: squat jump; SBJ: standing broad jump; VRT: variable resistance training; CRT: constant resistance training; ES: effect size.*

**Figure 2:**
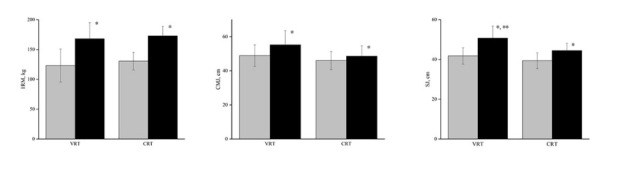
Mean difference in 1RM, CMJ, and SJ performance from pre-intervention (grey) to post-intervention (black) for each group. 1RM, 1-repetition maximum; CMJ, countermovement jump; SJ, squat jump; VRT, variable resistance training; CRT, constant resistance training. *Significant difference within-group. **Significant difference between-group.

No significant group × time interactions were observed for the back squat 1RM (F = 0.209, *p* = 0.653). There was a highly significant main effect over time (F = 219.731, *p* < 0.001). Both groups exhibited a highly significant within-group improvement in the back squat 1RM (VRT = 44.91 kg [95% CI, 33.42–56.39 kg], *p* < 0.001) (CRT = 42.22 kg [95% CI, 38.83–45.61 kg], *p* < 0.001). In addition, both groups showed a significant within-group improvement in the back squat 1RM mid-intervention (VRT = 20.18 kg, +16.4%) (CRT = 15.44 kg, +11.8%). There was no significant difference between the VRT and CRT groups in the back squat 1RM post-intervention (F = 0.218, *p* = 0.646) (Figure 2).

For the CMJ test, no significant group × time interactions were observed (F = 4.159, *p* = 0.056). There was a highly significant main effect over time (F = 23.23, *p* < 0.001). Both groups exhibited a significant within-group improvement in the CMJ (VRT = 6.33 cm [95% CI, 2.99–9.66 cm], *p* = 0.002) (CRT = 2.56 cm [95% CI, 0.51–4.62 cm], *p* = 0.02). No significant difference between groups were found post-intervention (F = 4.022, *p* = 0.06) (Figure 2). For the SJ test, highly significant group × time interactions were observed (F = 8.829, *p* = 0.008). The SJ improved significantly more following VRT compared to CRT (F = 7.403, *p* = 0.014) (Figure 2). Both groups exhibited a highly significant within-group improvement in the SJ (VRT = 8.95 cm [95% CI, 7.11–10.78 cm], *p* < 0.001) (CRT = 5.07 cm [95% CI, 3.04–7.10 cm], *p* < 0.001).

No significant group × time interactions were observed for the SBJ (F = 0.4, *p* = 0.535). There was a highly significant main effect over time (F = 9.552, *p* = 0.006). The VRT group exhibited a significant within-group improvement in the SBJ (7.91 cm [95% CI, 0.98–14.84 cm], *p* = 0.029). No significant within-group difference in the SBJ was observed following CRT (5.22 cm [95% CI, -1.90–11.57 cm] , *p* = 0.094). There was no significant difference in the SBJ between the VRT and CRT groups post-intervention (F = 2.931, *p* = 0.104).

No significant group × time interactions were observed for 10 m (F = 0.002, *p* = 0.965) and 20 m (F = 0.277, *p* = 0.605) sprint performance. No significant within- or between-group differences were found with respect to 10 and 20 m sprint performance (all, *p* > 0.05).

## Discussion

This study compared the effects of 8-week VRT and CRT, within a complex training program, on strength and power performance in well-trained collegiate basketball players. Contrary to our hypothesis, both groups showed similar improvements in maximum strength. As expected, the VRT group showed a significant increase in the SJ and a tendency toward a higher CMJ compared with the CRT group. However, no significant differences were found in respect to the SBJ and sprint performance between groups.

Considering maximum strength, no significant difference was observed between groups. This suggests that VRT provides similar maximum strength adaptations compared with CRT following an 8-week training intervention. Our results are in agreement with a meta-analysis that included studies with interventions of ≥7 weeks (Nilo dos Santos et al., 2018). Interestingly, several studies have reported significantly greater strength gains following VRT of up to a maximum of a 6-week intervention (Ataee et al., 2014; Joy et al., 2016; Katushabe and Kramer, 2020). One possible explanation for the disputing results could be the different methodologies used in VRT. Since different VRT strategies provided dissimilar mechanical effects (Frost et al., 2010), it may underlie different long-term training adaptations. Additionally, training periodization might be another consideration for the maximum strength gains following VRT. In general, neural adaptation is considered the primary factor for strength improvement in the early stages of a training program (Folland and Williams, 2007). In the current study, the back squat 1RM was reassessed mid-intervention. A greater improvement was found following VRT (+16.4%) compared to CRT (+11.8%) when pre-intervention scores were considered. By contrast, both groups presented a similar improvement (VRT +17%, CRT +18.3%) in the 1RM from 4-8 weeks. This reveals that VRT may be more prone to greater strength adaptation in the early phase of the intervention. In addition, our results indicate a highly significant within-group improvement in the back squat 1RM in both groups following an 8-week training intervention. These superior increases may be probably explained by the players’ background. Since our participants only had recreational resistance training experience prior to the study, significant improvements in maximum strength would be expected.

Our findings regarding the CMJ and SJ output showed significant within-group improvements in both groups. Our results concur with previous studies (Andersen et al., 2015; Joy et al., 2016; Katushabe and Kramer, 2020). Andersen et al. (2015) reported that VRT and CRT groups significantly increased the CMJ in two modes (starting depth of a 60° and a 90° knee angle) after a 10-week intervention with two sessions per week, whereas no significant difference was observed between groups. Joy et al. (2016) found a greater improvement in the vertical jump in the VRT group compared with the CRT group over a 5-week training intervention (one session per week). It is important to emphasize that the magnitude of vertical jump height improvements observed in our study (+5.6–21.4%) was much larger than of those only programming strength training (+4.1– 13.7%) (Andersen et al., 2015; Joy et al., 2016; Katushabe and Kramer, 2020). Considering the potential power benefits obtained when utilizing complex training in team sports (Cormier et al., 2020), this observation was expected. To the best of our knowledge, this is the first investigation comparing longitudinal power adaptations to VRT and CRT within a complex training program. The present study demonstrated a significant improvement in the SJ (*p* = 0.014, ES = 1.17) in the VRT group compared with the CRT group. For the CMJ, despite no significant between-group difference (*p* = 0.06) was observed, the ES following VRT (0.82) was larger than that following CRT (0.43). These results seem to suggest that PAPE is much larger following VRT than CRT. Although no electromyographic and velocity data were collected during back squat performance, some plausible explanations may be considered. One reasonable explanation is that lower loading at the initial concentric phase in VRT may allow a greater velocity in the biomechanically disadvantage position (i.e., sticking point) (Kompf and Arandjelović, 2016), and therefore theoretically leading to faster muscle fiber adaptations. Additionally, higher loading at the end of the concentric phase in VRT allows players to perform near maximum capacity, which may potentially increase the phosphorylation of regulatory light chains and the recruitment of higher order motor units (Tillin and Bishop, 2009). These neuromuscular responses possibly allow for a greater PAPE in a complex pair.

There was an improvement in SBJ performance in the CRT group following the 8-week intervention, although not statistically significant (*p* = 0.094). Our result is consistent with Freitas et al. (2019) who reported a moderate ES for the SBJ in a modified complex training group after a 6-week, 2 sessions per week intervention. In addition, the current study showed a significant within-group improvement in the SBJ in the VRT group. Two studies investigated acute PAPE effects of VRT on SBJ performance across four complex pairs (Seitz et al., 2016; Strokosch et al., 2018), and +3.8%–6% improvements compared to baseline were reported. Although we used similar loading as in those two studies, only a + 2.9% within-group improvement was observed. Based on the fatigue/potentiation relationship, the magnitude and the temporal profile of potentiation are affected by the intensity and volume used in the conditioning activity (Tillin and Bishop, 2009). Specifically, both studies (Seitz et al., 2016; Strokosch et al., 2018) used 2-repetition back squats with 90 s rest intervals intra complex pairs, while we used 3–6 repetitions with 3 min rest intervals intra complex pairs. Despite the fact that the repetitions performed in the current study might be fatiguing, the recovery was twofold compared to the above-mentioned studies. One possible explanation related to the discrepant results might be the subjects characteristics. In addition, the SBJ complex pairs in the current study were fixed in the third or fourth set in each session. Given that a decreased SBJ performance as the sets continued was found (Seitz et al., 2016; Strokosch et al., 2018), it is speculated that fatigue might have accumulated after performing multiple sets, thereby attenuating the PAPE effects.

Sprint performance did not change significantly in either group. To the authors’ knowledge, only two studies have investigated the effects of VRT on sprint performance, which demonstrated no improvement in 20 m (Loturco et al., 2020) and 40 m (Katushabe and Kramer, 2020) sprint performance. In addition, one study where CRT was performed within a complex training program also showed no significant changes in sprint performance, despite the fact that the magnitude of improvement in the back squat 1RM was approximately +50% (Kobal et al., 2017). It seems that improvements in maximum strength may not be the prerequisite for the development of sprint performance within the complex pair. A further consideration for optimization of PAPE between the conditioning activity and subsequent power performance is the biomechanical similarity (Tillin and Bishop, 2009). The study using a sled pull (Seitz et al., 2017) as a conditioning activity demonstrated acute significant improvement in sprint performance. From a mechanical perspective, the sled pull highlights horizontal force production, which is kinematically correlated to sprint performance. By performing such a conditioning activity, the individual is more likely to activate the potential mechanisms of PAPE specifically associated with sprinting (Tillin and Bishop, 2009).

Although the current study expands knowledge on the longitudinal effects of VRT within complex training on power performance, some limitations need to be acknowledged and addressed. The major limitation of the study was that a fixed contribution of variable resistance was used in the VRT group. As strength of players greatly increased in the 2^nd^ four weeks, the actual variable resistance percentage of 1RM was lower than that in the 1^st^ four weeks, thereby possibly attenuating the VRT training effect. In addition, although participants in the current study were well-trained collegiate basketball players, they only had recreational resistance training experience prior to the intervention. Thus, we used the 3RM test to prevent possible injury to the participants, which may not have accurately determined the 1RM value.

## Conclusions

The main findings of the present study demonstrated that performing VRT within a complex training program was more effective to enhance vertical and horizontal jump performance, especially for the squat jump. In addition, a similar improvement in maximal strength was observed in both training modalities following 8 weeks of training. However, we found greater strength adaptations in the 1^st^ 4-week training period in the VRT group. Thus, strength and conditioning practitioners could consider to program VRT into a short-term training period to overcome the plateau that occurs in strength progress. Additionally, the lack of biomechanical specificity during the current study may have compromised the development of sprint performance. Practitioners should consider using a kinematically similar conditioning activity if the aim is to improve sprint capacity.
